# Urethral Stricture with a Miniaturised (22 Fr) Versus Standard (26 Fr) Resectoscope Sheath During Thulium Fibre Laser Enucleation of the Prostate: A Prospective, Two-Centre Comparative Cohort Study

**DOI:** 10.3390/jcm15197724

**Published:** 2026-10-07

**Authors:** İbrahim Buldu, Mustafa Bilal Hamarat, Burak Yılmaz, Taha Emre Aydın, Hakan Hakkı Taşkapu

**Affiliations:** 1 Department of Urology, Medical Faculty, KTO Karatay University, Konya 42100, Turkey; 2 Department of Urology, Konya City Hospital, University of Health Sciences, Konya 42020, Turkey; mustafabilal.hamarat@sbu.edu.tr (M.B.H.); drburakyilmaz@hotmail.com (B.Y.); tahaemreaydn@gmail.com (T.E.A.); 3 Department of Urology, Medical Faculty, Necmettin Erbakan University, Konya 42080, Turkey; drtaskapu@hotmail.com

**Keywords:** BPH, prostate, MiLEP, AEEP, ThuFLEP, urethral stricture, resectoscope size

## Abstract

**Objectives:** We aimed to compare the observed rates of postoperative urethral stricture between patients undergoing thulium fibre laser enucleation of the prostate (ThuFLEP) using a 22 Fr miniaturised instrument strategy and those treated using a standard 26 Fr strategy. **Methods:** This prospective, two-centre comparative cohort study included 208 patients with benign prostatic hyperplasia and a prostate volume of less than 150 mL who underwent thulium fibre laser enucleation of the prostate (ThuFLEP) between 2024 and 2026. Patients were grouped according to the resectoscope sheath used at each centre: Group 1 comprised 99 patients treated with a 22 Fr miniaturised sheath and Group 2 comprised 109 patients treated with a standard 26 Fr sheath. The primary endpoint was the development of postoperative urethral stricture. Patient demographics and preoperative and postoperative clinical outcomes were also recorded. **Results:** Demographic and operative parameters were comparable between the groups. No significant difference was observed in mean prostate volumes (68.8 ± 34.1 vs. 70.8 ± 25.7 mL). Although the enucleation efficiency (g/min) was numerically higher in Group 1, the difference did not reach statistical significance (2.2 ± 1.1 vs. 1.8 ± 0.6; *p* = 0.082). Postoperative urethral stricture was observed in 2 patients (2.0%) in Group 1 and in 10 patients (9.2%) in Group 2. The incidence of stricture was significantly higher in Group 2 (*p* = 0.036). **Conclusions:** The 22 Fr ThuFLEP strategy was associated with a lower observed urethral stricture rate. However, the independent effect of sheath diameter could not be separated from centre- and surgeon-related factors. Further randomised studies are required to confirm these findings.

## 1. Introduction

Benign prostatic hyperplasia (BPH) is one of the most common causes of reduced quality of life and lower urinary tract symptoms (LUTS) in men over the age of 50. Anatomical endoscopic enucleation of the prostate (AEEP) is an established, guideline-supported surgical treatment for BPH. Since it was first described in 1998, AEEP has proven to be an effective and safe method for treating prostates of all sizes [1]. TFL technology allows continuous-wave energy delivery for haemostasis and pulsed energy delivery for tissue enucleation [2,3].

As with other transurethral surgical procedures, urethral stricture may occur in 2–10% of cases following AEEP [4]. Standard resectoscopes currently in use have a diameter of 26 Fr. However, in a study by Bhat and colleagues, it was observed that 13% of men had a urethral diameter of less than 26 Fr [5]. In recent years, the miniaturisation of resectoscope diameters has been aimed at reducing various urethral complications, particularly urethral stricture [6,7]. In this study, we investigated whether the use of a 22 Fr miniaturised resectoscope sheath during ThuFLEP was associated with a lower incidence of postoperative urethral stricture compared with a standard 26 Fr sheath.

## 2. Materials and Methods

This two-centre comparative cohort study was conducted between December 2024 and May 2026 at KTO Karatay University Medova Hospital and the University of Health Sciences Konya City Hospital. Patients with a prostate volume of less than 150 mL were prospectively enrolled in sequential order. At Medova Hospital, all ThuFLEP procedures were performed using a 22 Fr resectoscope (Wurmlingen, Germany) by a single experienced surgeon who had previously performed more than 500 procedures. At Konya City Hospital, all procedures were performed using a 26 Fr resectoscope (Knittlingen, Germany) by another experienced surgeon who had previously performed more than 300 procedures. Group allocation was determined by the instrument system used at each centre, without randomisation. To reduce procedural variability, both centres used an en-bloc enucleation technique with early apical release, the same laser settings, and the same postoperative catheter size. Each sheath size was used exclusively at one centre by a single surgeon. Consequently, sheath size, centre, and surgeon were completely confounded.

The study included patients with lower urinary tract symptoms due to BPH, those who had suffered recurrent urinary tract infections, those with recurrent haematuria, those who had developed hydronephrosis in the upper urinary tract, and those who had not responded to medical treatment.

Patients with a history of prostate surgery, previous interventions for urethral stricture, neurogenic bladder, bladder stones, prostate cancer, or pelvic radiotherapy were excluded from the study. Patients who required intraoperative urethral dilatation or urethrotomy for severe urethral stricture were also excluded. Severe urethral narrowing was operationally defined as narrowing that prevented passage of the resectoscope used at the respective centre and required dilatation or urethrotomy. Patients requiring these interventions were excluded. Mild or moderate narrowing did not preclude inclusion if resectoscope passage was possible without either intervention and the remaining eligibility criteria were met. Patients excluded because of intraoperative urethrotomy did not undergo postoperative study follow-up.

Preoperative assessment included digital rectal examination, urinalysis, prostate volume measurement, prostate-specific antigen (PSA) testing, maximum urinary flow rate (Qmax) measurement, post-void residual urine volume (PVR) measurement, and assessment using the International Prostate Symptom Score (IPSS). Patient demographics and perioperative and postoperative outcomes were also evaluated. Preoperative Qmax was not measured in patients with an indwelling urinary catheter. Unavailable Qmax measurements, whether at baseline or during follow-up, were treated as missing and were not assigned a value of zero. Qmax comparisons were based on patients with available measurements at the corresponding assessment. Enucleation efficiency (g/min) was calculated for each patient by dividing the weight of the enucleated prostatic tissue, measured in the operating room, by the enucleation time (min).

Follow-up assessments were scheduled at 1, 3, 6, and 12 months postoperatively and included uroflowmetry, PVR measurement, and IPSS assessment. Urethral stricture was suspected when Qmax was below 15 mL/s or when the uroflowmetry curve showed a pattern suggestive of urethral stricture. A suggestive uroflowmetry pattern was defined as a flattened or plateau-shaped curve with prolonged voiding and persistently low flow. In these cases, the diagnosis was confirmed by urethrography or cystoscopy. Urethral stricture was recorded only after anatomical confirmation.

All procedures were performed using a 60 W thulium fibre laser (TFL; Quanta System, Italy) with a 550 μm fibre. The laser settings were standardised at 2 J and 30 Hz for all patients. Group 1 used a 22 Fr continuous-flow resectoscope (Tontarra, Wurmlingen, Germany) with a 2.9 mm telescope, whereas Group 2 used a 26 Fr continuous-flow resectoscope (Richard Wolf, Knittlingen, Germany) with a 5 mm telescope. Both telescopes had a 30° viewing angle. For morcellation, a 22 Fr MiniLEP nephroscope was used in Group 1 and a 26 Fr Richard Wolf nephroscope in Group 2. The same morcellator system, the Hawk Shaver System, was used in both groups. Thus, the nephroscopes used for morcellation differed between groups, while the tissue morcellator system was the same.

All procedures at both centres were performed using an anterior (top-down) en-bloc enucleation approach with early apical release. Thus, the overall direction of enucleation was consistent across the two surgeons and treatment groups. At the end of each procedure, a 22 Fr three-way Foley catheter was inserted, and continuous bladder irrigation was initiated.

Continuous variables are presented as mean ± standard deviation, and categorical variables are presented as counts and percentages. Between-group comparisons were performed using the Mann–Whitney U test for continuous variables and the chi-square test or Fisher’s exact test, as appropriate, for categorical variables. The unadjusted odds ratio (OR) for postoperative urethral stricture was calculated from the 2 × 2 contingency table, using the 22 Fr group as the reference. The 95% confidence interval for the odds ratio was calculated using the log-Wald method, based on the numbers of patients with and without urethral stricture in each group. No covariate adjustment was applied. No formal a priori sample-size calculation was performed. The sample size was determined by the number of consecutive eligible patients enrolled during the study period. Accordingly, this prospective comparative cohort study should be considered exploratory. ChatGPT (GPT-5.2, OpenAI) was used to assist with statistical calculations and interpretation.

All procedures were performed in accordance with the 1964 Declaration of Helsinki and its subsequent amendments. The study was approved by the institutional ethics committee (approval no. 22.11.2024-33429).

## 3. Results

A total of 234 patients underwent ThuFLEP during the study period. Twelve patients were excluded based on preoperative criteria: previous surgery (*n* = 5), neurogenic bladder (*n* = 3), or prostate cancer (*n* = 4). An additional 14 patients were excluded because they required intraoperative urethrotomy for a pre-existing urethral stricture that prevented resectoscope passage: 3 patients in the 22 Fr group and 11 patients in the 26 Fr group. The final analytical cohort comprised 208 patients, including 99 in the 22 Fr group and 109 in the 26 Fr group.

The remaining 208 patients were included in the final analysis, comprising 99 in Group 1 and 109 in Group 2. The mean follow-up duration was 9.5 months. The mean age was 67.4 ± 8.7 years in Group 1 and 69.6 ± 6.3 years in Group 2. No significant between-group differences were observed in prostate volume (68.8 ± 34.1 vs. 70.8 ± 25.7 mL) or total PSA levels (2.9 ± 2.5 vs. 3.1 ± 2.3 ng/mL). Baseline demographic and clinical characteristics are summarised in Table 1. The proportion of smokers was significantly higher in Group 2 than in Group 1 (31.3% vs. 18.2%; *p* = 0.030). Preoperative Qmax was significantly higher in Group 1 than in Group 2 (8.34 ± 3.5 vs. 6.9 ± 2.3 mL/s; *p* = 0.018), whereas preoperative IPSS was similar between the groups (20.4 ± 4.9 vs. 23.4 ± 4.2). The proportion of patients with a preoperative urinary catheter did not differ significantly between the groups (15.2% vs. 15.6%).

The mean enucleation time was 42.7 ± 20.5 min in Group 1 and 39.8 ± 14.0 min in Group 2, with no statistically significant difference. Enucleation efficiency was numerically higher in Group 1, although the difference did not reach statistical significance (2.2 ± 1.1 vs. 1.8 ± 0.6; *p* = 0.082). Morcellation time and efficiency were also comparable between the groups (Table 2). Intraoperative optical malfunction occurred in three patients in Group 1 and in none in Group 2. All optical malfunctions occurred during procedures in patients with prostate volumes greater than 100 mL.

No significant between-group differences were observed in the length of hospital stay (1.3 ± 0.5 vs. 1.4 ± 0.6 days) or postoperative catheterisation duration (1.9 ± 0.7 vs. 2.0 ± 0.6 days). Transfusion rates were also similar (3.0% vs. 3.6%). Postoperative Qmax was comparable between Group 1 and Group 2 (17.0 ± 5.7 vs. 16.9 ± 4.6 mL/s). However, postoperative PVR was significantly higher in Group 2 than in Group 1 (29.9 ± 22.6 vs. 25.8 ± 19.6 mL; *p* = 0.012) (Table 3).

Postoperative urethral stricture was detected in 2/99 patients (2.0%) in the 22 Fr group and 10/109 patients (9.2%) in the 26 Fr group. The unadjusted OR for the 26 Fr group relative to the 22 Fr group was 4.90 (95% CI, 1.05–22.94; *p* = 0.036). The wide confidence interval reflects substantial imprecision, with only 12 events observed. All confirmed strictures were located in the bulbar urethra.

## 4. Discussion

Transurethral procedures are the most commonly performed surgical interventions for benign prostatic hyperplasia (BPH). Among these, anatomical endoscopic enucleation of the prostate (AEEP) is an established treatment option with demonstrated efficacy and safety. Thulium fibre laser (TFL) technology offers favourable haemostatic properties and pulsed energy delivery characteristics. In developed countries, iatrogenic injury accounts for 32–79% of urethral strictures, with transurethral procedures contributing to approximately 41% of these cases [8].

In this prospective, two-centre comparative cohort study, the 22 Fr group had a lower observed postoperative urethral stricture rate than the 26 Fr group (2.0% versus 9.2%; *p* = 0.036). However, sheath size was completely confounded with centre and surgeon, as each sheath size was used exclusively at one centre by a single surgeon. Although both surgeons were experienced and used the same overall enucleation approach and laser settings, these similarities do not exclude potential differences in surgical handling, patient selection, or institutional practices. Accordingly, the observed difference cannot be independently attributed to sheath diameter and should be interpreted as an association within the treatment settings studied rather than as evidence of a causal effect of sheath miniaturisation.

Urethral stricture is a late complication of transurethral prostate surgery that may develop following mucosal injury and subsequent spongiofibrosis in the anterior urethra. Reported stricture rates after endoscopic enucleation for BPH generally range from 2% to 10% [9]. Conventional continuous-flow resectoscopes commonly have sheath diameters of 26 Fr or greater, reflecting design requirements associated with transurethral resection of the prostate (TURP), including accommodation of the resection loop. However, the male urethra does not invariably permit atraumatic passage of instruments of this size. Approximately 13% of men have a urethral calibre below 26 Fr [5]. In these patients, forceful instrument insertion or additional urethral manipulation, including dilatation or urethrotomy, may contribute to mucosal injury and subsequent stricture formation. The rationale for instrument miniaturisation is to accommodate the natural urethral calibre and reduce mechanical pressure and potential ischaemic injury during instrumentation.

The relationship between instrument diameter and urethral stricture has been investigated across different surgical modalities. In a retsrospective study of monopolar TURP, Günes et al. reported that reducing the sheath diameter from 26 Fr to 24 Fr significantly decreased the rate of bulbar urethral stricture from 11.4% to 2.9% [10]. In laser enucleation, Özsoy et al. prospectively compared 22 Fr and 26 Fr sheaths during thulium laser enucleation [11]. No patients in the 22 Fr MiLEP group required intraoperative urethrotomy or meatotomy, whereas these interventions were required in 12.5% of patients (*n* = 10) in the standard 26 Fr group. At six months, urethral stricture rates were 2.9% and 5.0%, respectively, although the difference was not statistically significant. The authors suggested that the involvement of a single highly experienced surgeon may have reduced variability in urethral manipulation. Our study was conducted at two centres by surgeons experienced in AEEP and demonstrated a significantly lower stricture rate with the miniaturised sheath. Our findings are consistent with the hypothesis that smaller instruments may be associated with lower stricture rates; however, the complete confounding of sheath size with centre and surgeon prevents independent attribution of the observed difference to instrument diameter. Randomised evidence does not uniformly favour smaller instruments. Dean et al. compared 24 Fr and 28 Fr sheaths during HoLEP and found no significant differences in functional outcomes or complications at three months, while some immediate recovery outcomes favoured the 28 Fr system. Although differences in laser modality and sheath sizes limit direct comparison with our study, these findings caution against assuming that miniaturisation necessarily improves postoperative outcomes [12].

Whether miniaturisation compromises surgical efficacy and safety remains one of the most widely debated issues in the literature. There is concern that reduced irrigation flow through smaller sheaths may impair visibility and prolong enucleation. In our study, however, mean enucleation time was comparable between the 22 Fr and 26 Fr groups (42.7 ± 20.5 vs. 39.8 ± 14.0 min; *p* = 0.381), as was morcellation time. Enucleation efficiency was numerically higher in the 22 Fr group (2.2 ± 1.1 vs. 1.8 ± 0.6 g/min), although the difference was not statistically significant (*p* = 0.082). These findings are consistent with those reported by Figueiredo et al. [13] and suggest that a 22 Fr sheath can provide adequate operating conditions for efficient enucleation. En-bloc dissection along the appropriate surgical plane and the haemostatic properties of TFL may help maintain visibility despite the smaller sheath diameter. Nevertheless, sheath diameter is unlikely to be the sole determinant of stricture risk. Longer operative duration, intraoperative meatotomy or other urethral complications, and smaller prostate volume have also been associated with stricture risk in some HoLEP cohorts [14].

Additional observational evidence suggests that patient characteristics and postoperative complications may also be associated with stricture formation. In a retrospective analysis of 246 patients undergoing thulium laser or bipolar enucleation, Hou et al. reported urethral strictures in 9.3% of patients; smaller prostate volume, lower enucleated tissue weight, failure of a trial without catheter, and postoperative urinary tract infection were associated with stricture formation [15]. In a large retrospective cohort comparing monopolar TURP with open simple prostatectomy, Golomb et al. found that older age and greater comorbidity burden were associated with postoperative urethral stricture [16]. Although differences in surgical procedures and outcome ascertainment limit direct comparison with our cohort, these studies underscore the need to consider factors beyond instrument diameter. The relationships between postoperative complications and stricture formation were not evaluated in our study, limiting interpretation of the observed between-group difference.

The length of hospital stay (1.3 ± 0.5 vs. 1.4 ± 0.6 days) and postoperative catheterisation duration (1.9 ± 0.7 vs. 2.0 ± 0.6 days) were similar in the 22 Fr and 26 Fr groups. Postoperative Qmax was also comparable (17.0 ± 5.7 vs. 16.9 ± 4.6 mL/s). Postoperative PVR was significantly higher in the 26 Fr group than in the 22 Fr group (29.9 ± 22.6 vs. 25.8 ± 19.6 mL; *p* = 0.012). However, the absolute difference was small, and its clinical relevance remains uncertain. Urethral oedema or instrumentation-related injury may have contributed to this difference, although these mechanisms were not directly assessed in the present study. Therefore, this finding should not be interpreted as evidence of superior bladder emptying with the 22 Fr strategy.

Intraoperative optical malfunction occurred in three patients in the 22 Fr group (3/99, 3.0%) and in none in the 26 Fr group (0/109). All three events occurred in patients with prostate volumes greater than 100 mL. These findings should be considered descriptive and exploratory, as the small number of events and differences between instrument systems preclude attributing the malfunctions to telescope diameter or establishing an association with prostate volume. In the multicentre MiLEP series reported by Cindolo et al., no resectoscope or optical failures were reported [17]; however, potential differences in outcome definitions and reporting practices limit direct comparison. These observations warrant further evaluation of instrument durability in miniaturised systems but do not support specific recommendations based on telescope diameter or prostate volume.

The principal limitation is that sheath size, surgeon, centre, telescope diameter, and instrument system were not independently varied, preventing isolation of the effect of sheath diameter. Shared operative techniques and laser settings do not eliminate differences in surgical handling, patient selection, or perioperative care. The unadjusted analysis also leaves potential confounding by smoking and baseline Qmax; the latter was unavailable in catheterised patients.

Exclusion of patients requiring intraoperative urethrotomy was more frequent in the 26 Fr group (*n* = 11) than in the 22 Fr group (*n* = 3), potentially introducing selection bias related to urethral calibre and instrument size. Postoperative outcomes were unavailable for these patients, precluding an outcome-based sensitivity analysis.

No a priori sample-size calculation was performed, and only 12 strictures occurred. The wide confidence interval around the unadjusted OR of 4.90 (95% CI, 1.05–22.94) indicates substantial statistical imprecision. Furthermore, the mean follow-up of 9.5 months may not capture late strictures. Group-specific visit completion and individual stricture diagnosis times were unavailable, preventing assessment of comparable surveillance and time-to-event analysis. The reported proportions therefore reflect events detected during available follow-up rather than standardised 12-month risks.

These findings are exploratory and hypothesis-generating. Adequately powered randomised studies comparing both sheath sizes within participating centres and surgeons, with longer follow-up, are required.

## 5. Conclusions

In this prospective two-centre cohort, the 22 Fr instrument strategy was associated with a lower observed rate of postoperative urethral stricture. Because treatment group was inseparable from centre, surgeon, and instrument system, randomised studies with longer follow-up are required to determine the independent effect of sheath diameter.

## Figures and Tables

**Table 1:** Demographic and preoperative data of patients.

	Group 1 (22 Fr)	Group 2 (26 Fr)	*p* Value
Number of patients (N)	99	109	
Age (year)	67.4 ± 8.7	69.6 ± 6.3	0.130
BMI	29.7 ± 4.4	28.6 ± 3.9	0.490
Prostate volume (mL)	68.8 ± 34.1	70.8 ± 25.7	0.152
Total PSA (ng/mL)	2.9 ± 2.5	3.1 ± 2.3	0.292
Diabetes	28 (28.3%)	27 (24.8%)	0.566
Cardiovascular disease	76 (76.7%)	80 (73.4%)	0.412
Smoking	18 (18.2%)	34 (31.3%)	**0.030**
IPSS	20.4 ± 4.9	23.4 ± 4.2	0.106
Qmax (mL/s)	8.34 ± 3.5	6.9 ± 2.3	**0.018**
PVR (mL)	176.5 ± 110.6	155.4 ± 80.2	0.569
Indwelling catheter prior to surgery	15 (15.2%)	17 (15.6%)	0.929

Bold indicates statistically significant results.

**Table 2:** Perioperative data for patients.

	Group 1 (22 Fr)	Group 2 (26 Fr)	*p* Value
Duration of enucleation	42.7 ± 20.5	39.8 ± 14.0	0.381
Enucleation efficacy (g/min)	2.26 ± 1.19	1.86 ± 0.6	0.082
Morcellation time (min)	10.1 ± 5.2	10.2 ± 3.4	0.063
Morcellation efficacy (g/min)	7.6 ± 3.5	7.5 ± 2.9	0.997
Technical problem (optical malfunction)	3 (3%)	0 (0%)	0.106

**Table 3:** Patients’ postoperative data.

	Group 1 (22 Fr)	Group 2 (26 Fr)	*p* Value
Hospitalisation (day)	1.3 ± 0.5	1.4 ± 0.6	0.684
Transfusion rate	3.0 (3%)	4 (3.6%)	1.000
Postoperative catheterisation	1.9 ± 0.7	2.0 ± 0.6	0.479
Qmax	17.0 ± 5.7	16.9 ± 4.6	0.757
PVR	25.8 ± 19.6	29.9 ± 22.6	**0.012**
Postoperative urethral stricture	2 (2%)	10 (9.2%)	**0.036**

Bold indicates statistically significant results.

## Data Availability

The data supporting the findings of this study are available from the authors upon reasonable request.

## Abbreviations

AEEP: anatomical endoscopic enucleation of the prostate; ThuFLEP: thulium fibre laser enucleation of the prostate; TFL: thulium fibre laser; BPH: benign prostatic hyperplasia; MiLEP: miniaturised laser enucleation of the prostate; LUTS: lower urinary tract symptoms; Qmax: maximum urinary flow rate; PVR: post-void residual urine volume.
